# Expression Signatures of Long Noncoding RNAs in Left Ventricular Noncompaction

**DOI:** 10.3389/fcvm.2021.763858

**Published:** 2021-11-10

**Authors:** Qingshan Tian, Hanxiao Niu, Dingyang Liu, Na Ta, Qing Yang, Vikram Norton, Yujing Wu, Amit K. Maiti, Hao Wu, Zhenzhong Zheng

**Affiliations:** ^1^Department of Cardiology, The First Affiliated Hospital of Nanchang University, Nanchang, China; ^2^Department of Functional Examination, Shaanxi Provincial People's Hospital, Xi'an, China; ^3^Department of Cardiology, Jiangxi Provincial People's Hospital, Nanchang, China; ^4^Postoperative Cardiac Intensive Care Unit, Fuwai Hospital Chinese Academy of Medical Sciences, Shenzhen, China; ^5^Vascular Biology Program, Department of Surgery, Harvard Medical School, Boston Children's Hospital, Boston, MA, United States; ^6^Department of Emergency, The First Affiliated Hospital of Xi'an Jiaotong University, Xi'an, China; ^7^Mydnavar, Department of Genetics and Genomics, Troy, MI, United States

**Keywords:** lncRNAs, LVNC, mRNAs, miRNA targets, biomarker

## Abstract

Long noncoding RNAs have gained widespread attention in recent years for their crucial role in biological regulation. They have been implicated in a range of developmental processes and diseases including cancer, cardiovascular, and neuronal diseases. However, the role of long noncoding RNAs (lncRNAs) in left ventricular noncompaction (LVNC) has not been explored. In this study, we investigated the expression levels of lncRNAs in the blood of LVNC patients and healthy subjects to identify differentially expressed lncRNA that develop LVNC specific biomarkers and targets for developing therapies using biological pathways. We used Agilent Human lncRNA array that contains both updated lncRNAs and mRNAs probes. We identified 1,568 upregulated and 1,141 downregulated (log fold-change > 2.0) lncRNAs that are differentially expressed between LVNC and the control group. Among them, RP11-1100L3.7 and XLOC_002730 are the most upregulated and downregulated lncRNAs. Using quantitative real-time reverse transcription polymerase chain reaction (RT-QPCR), we confirmed the differential expression of three top upregulated and downregulated lncRNAs along with two other randomly picked lncRNAs. Gene Ontology (GO) and KEGG pathways analysis with these differentially expressed lncRNAs provide insight into the cellular pathway leading to LVNC pathogenesis. We also identified 1,066 upregulated and 1,017 downregulated mRNAs. Gene set enrichment analysis (GSEA) showed that G2M, Estrogen, and inflammatory pathways are enriched in differentially expressed genes (DEG). We also identified miRNA targets for these differentially expressed genes. In this study, we first report the use of LncRNA microarray to understand the pathogenesis of LVNC and to identify several lncRNA and genes and their targets as potential biomarkers.

## Introduction

Left ventricular noncompaction is a rare disease that is characterized by the failure of densification in the normal embryonic cardiac tissue. It can occur in isolation or association with congenital heart defects (CHDs), neuromuscular disorders, and systemic heart anomalies (1, 2). Morphological features of left ventricular noncompaction (LVNC) consist of deep trabecular recesses in the myocardial wall with noncompaction of the loosely interwoven meshwork in the left ventricular cavity (3). The typical clinical result is a triad of heart failure, arrhythmias, and systemic embolism (4).

Left ventricular noncompaction is a genetically heterogeneous disease (5) and is associated with genes and/or proteins that are involved in sarcomere (MYH7, ACTC, MYBPC3, TNNT2), cytoskeletal (ZASP, LMNA), and mitochondrial structure or function (TAZ) (6–9). However, using exome and mitochondrial DNA sequencing from myocardial tissue samples, Liu Z et al. identified mutations in 16S rRNA 2336T>C mitochondrial mutation but did not detect any pathogenic mutations in TNNT2 and MYBPC3 genes (10).

Long noncoding RNA molecules are >200 nt in length, similar to mRNA, but do not code for structural proteins. Rather, they modulate the expression of protein-coding genes (11). Particularly, relating lncRNA with disease pathogenesis has widespread implications. Long noncoding RNAs (LncRNAs) are easily targetable, and their expression can be controlled from external oligo DNA or RNA that can be administered through blood or tissue specifically (12). A disease development could be delayed or cured by externally manipulating their expressions. Such advantages recently gained specific interest in identifying disease-specific lncRNA (13, 14).

Numerous studies show that lncRNAs play a positive and negative role in the differentiation, development, and progression of many diseases such as cancer, neurodegenerative diseases, and heart diseases (13, 15–17). The role of lnCRNA in cardiac dysfunction is especially beginning to be elucidated (18, 19). However, the role of lncRNA in LVNC has not been reported. This study compared the lncRNA and mRNA expression in the blood of six LVNC patients with six healthy subjects as controls and correlated their association with LVNC. Identification of these differentially expressed lncRNAs and mRNA may provide essential targets to develop blood-based biomarkers and potential therapeutic interventions for treating patients with LVNC.

## Methods

### Patients and Healthy Subjects

The blood samples of LVNC patients and healthy subjects were obtained from May 2015 to October 2015 in the Department of Cardiology in the First Affiliated Hospital of Nanchang University. Six patients with LVNC were selected for the case-study group and six healthy subjects were selected as the control group. Clinical characteristics of LVNC patients and control subjects are presented in Table 1. Written informed consents were obtained from all patients and the study was approved by the Institutional Review Board (IRB) of the First Affiliated Hospital of Nanchang University. We followed all ethical compliance for the study.

**Table 1:** Clinical characteristics of study populations.

**Characteristic**	**LVNC** **(***n*** = 6)**	**Control** **(***n*** = 6)**
Age (y)	66.3 ± 8.43	66.3 ± 8.43
Gender (male/female)	3/3	3/3
LVEF (%)	36.7 ± 11.6	62.2 ± 5.4
**NYHA functional class at time of biopsy n (%)**		
I or II	2 (33%)	6 (100%)
III or IV	4 (67%)	0
Systolic BP (mmHg)	113 ± 12.7	133 ± 5.0
Diastolic BP (mmHg)	82 ± 8.1	79 ± 5.9
Plasma NT-proBNP levels (pg/mL)	>2,000 (100%)	<500(100%)
Thromboembolic events	0/6	0/6
Arrhythmias	5/6	0/6
Atrial fibrillation	1/6	0/6
**Medication (n/tn)**		
Loop diuretics	6/6	0/6
Spironolactone	6/6	0/6
Digitalis	5/6	0/6
ACEIs or ARBs	6/6	2/6
β-Blocker	1/6	1/6

### RNA Isolation and Array Hybridization

Blood samples from each patient and healthy subject were centrifuged at 1,000 rpm for 10 min to separate plasma from cells. They were then stored at −80°C. Total RNAs from each sample were prepared from blood cells with the RNA isolation kit (Qiagen, Canada) after removal of rRNA from a Eukaryotic mRNA Isolation Kit, Epicenter. Then, each sample was amplified and transcribed into fluorescent cRNA along the entire length of the transcripts, without 3' bias, utilizing a random priming method (Arraystar Flash RNA Labeling Kit, Arraystar). The labeled cRNAs were purified by RNeasy Mini Kit (Qiagen). The concentration and specific activity of the labeled cRNAs (pmol Cy3/μg cRNA) were measured by NanoDrop ND-1000 (Table 2). One microgram and each labeled cRNA was fragmented by adding 5 μl 10 × Blocking Agent and 1 μl of 25 × Fragmentation Buffer. The mixture was then heated at 60°C for 30 min. Finally, 25 μl 2 × GE Hybridization buffer was added to dilute the labeled cRNA. Fifty microliter of hybridization solution was dispensed into the gasket slide and assembled to the lncRNA expression microarray slide (Agilent lncRNA microarray chip (catalog no.: G4851C, Agilent technology) that contained 26,803 Entrez unique genes and 30,606 unique lncRNAs. Microarray hybridization was performed according to the Agilent One-Color Microarray-Based Gene Expression Analysis protocol (Agilent Technology) with minor modifications. The slides were incubated for 17 h at 65°C in an Agilent Hybridization Oven. The hybridized arrays were washed, fixed, and scanned using the Agilent DNA Microarray Scanner (part number G2505C).

**Table 2:** The primers for RT-qPCR.

**Target genes**	**Bidirectional primer sequence**	**Annealing temperature (^°^C)**	**Amplicon (bp)**
β-actin(H)	F:5′ GTGGCCGAGGACTTTGATTG3′	60	73
	R:5′ CCTGTAACAACGCATCTCATATT3′		
ENST00000423402	F:5′TAAGAAGCCGAATGAGGAAGAC3′	60	95
	R:5′GGTCAGTAAGGAAATCTGGAGG3′		
uc021ybu.1	F:5′TCGAGACCTTCAGCAATACCC3′	60	142
	R:5′CATACCACTCGTAGATGGGCAG3′		
uc002nqf.1	F:5′AGGTGACATCTGAGCTGGGTT3′	60	98
	R:5′TGTGCCTTGAGCAGATAGCC3′		
ENST00000550301	F:5′CAGCAAGAAGTGGTGGCTCTTC3′	60	92
	R:5′GACGGTTCAGTCCTGCCTCTAC3′		
ENST00000417844	F:5′ACCTTCTGGTGCATTTTAGCTT3′	60	105
	R:5′ATGAACAAGGCACTCAAGGAA3′		
ENST00000554022	F:5′GGTTCCCACATAACTCCACTCC3′	60	120
	R:5′CTTTCTTCCCAAACTGAGCCG3′		

### Validation of the Differentially Expressed LncRNAs by Quantitative Real-Time PCR (qRT-PCR)

RT-QPCR was used to validate the microarray lncRNA expression among the six LVNC and six healthy patients. The primer sequences of β-actin (H, ENST00000423402, uc021ybu.1, uc002nqf.1, ENST00000550301, ENST00000417844, and ENST00000554022 were shown in Table 2. The primers used for RT-QPCR were designed and synthesized by Invitrogen (Shanghai, China). β-actin was used as an internal control (housekeeping gene). RT-QPCR was performed using GeneAmp PCR System 9700 (Applied Biosystems). The PCR reaction conditions were an initial denaturation at 95°C for 10 min, followed by 40 PCR cycles at 95°C for 10 s, and 60°C for 60 s. The products were amplified for 10 min by slowly heating them from 60 to 99°C after the extension. The target (lncRNA or gene) and housekeeping genes for each sample were used for Real-time PCR reactions. The target gene/lncRNA concentration of each sample was divided by the concentration of the housekeeping gene to obtain the relative concentration of the target gene for each sample. The amount of target sequence for the sample was derived from the standard curve throughout the experiment.

### LncRNA Microarray Statistical Analysis

Agilent Feature Extraction software (version 11.0.1.1) was used to analyze acquired array images. Quantile normalization and subsequent data processing were performed by using the GeneSpring GX v12.1 software package (Agilent Technologies). After quantile normalization of the raw data, lncRNAs and mRNAs which had flags in at least six out of 12 samples in Present or Marginal (“All Targets Value”) were chosen for further data analysis. Differentially expressed lncRNAs and mRNAs with statistical significance between the two groups of healthy and LVNC patients were identified through *p*-value or FDR filtering. Differentially expressed lncRNAs and mRNAs between the two samples were identified through Fold Change filtering. Hierarchical Clustering and combined analysis were performed and the differences between mRNAs were analyzed to infer their role in cellular pathway using Gene Ontology (GO) and KEGG.

### Gene Set Enrichment Analysis (GSEA)

Gene set enrichment analysis (GSEA) is essentially done as described by Subramanian et al. (20). The top 1,500 differentially expressed genes (DEG) are used with the Hallmark Gene Set database. Analysis was done with a “weighted” scoring scheme with “no collapse” as the main parameters. This analysis picked 13 enriched pathways. Among these pathways, the G2M transition, estrogen response, and inflammatory pathways had the highest enrichment score and were studied further. Individual gene expression of GSEA significant genes in these pathways across the LVNC and healthy subjects are performed in R package with “plot” and “bar plot” function.

### Determination of Biological Functions and Identification of Targeted MiRNA

Various biological functions and interactome analysis of DEG are determined by the use of FUNRICH software (21). The top 500 DEGs are used as input to carry out the analysis.

Information about biological pathways and target miRNA for these DEGs are deduced by using EnrichR software (22–24). The top 500 DEGs are also used as input to identify pathways and miRNA.

## Result

### Microarray Expression Analysis

**Figure 1:**
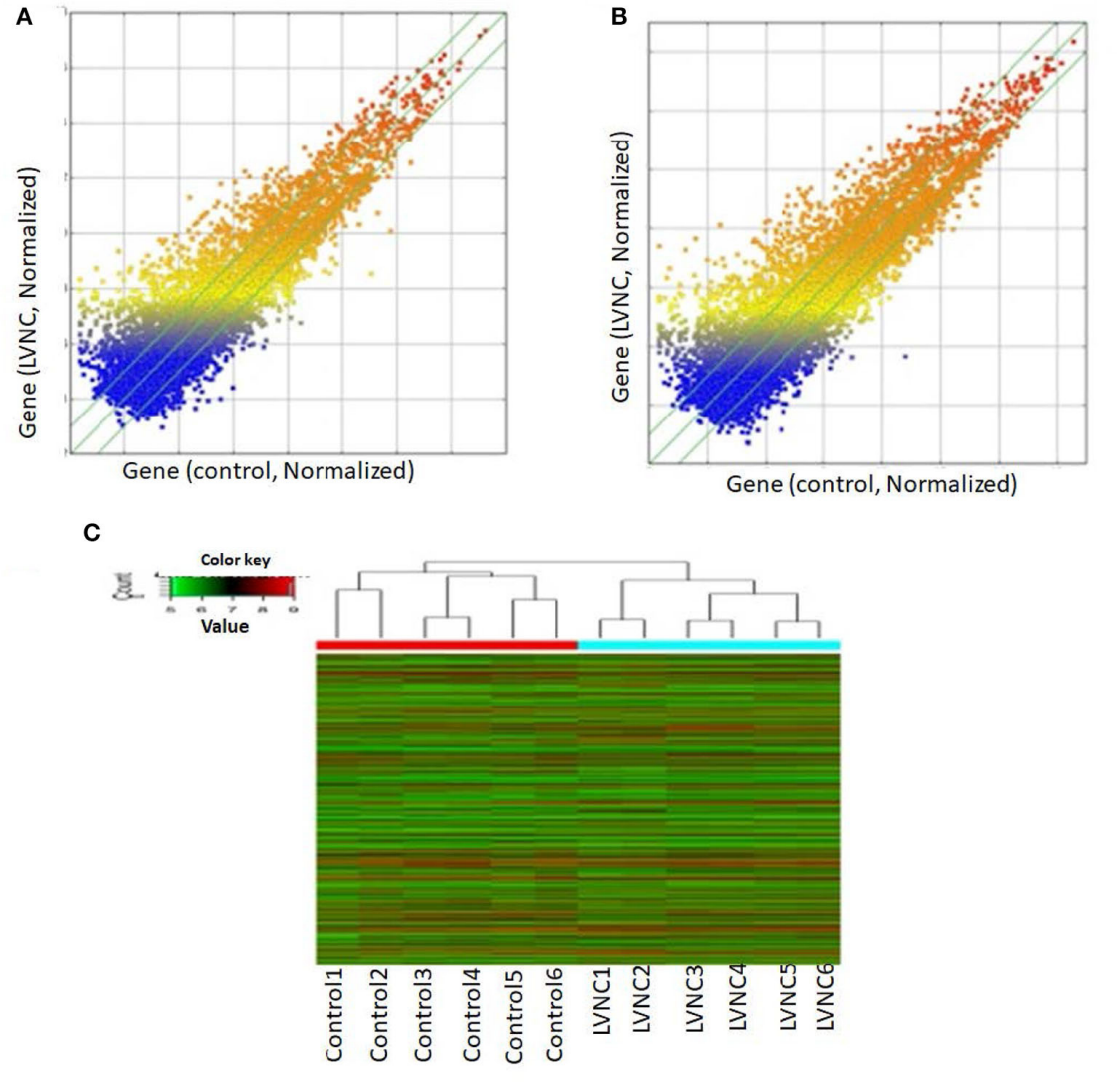
Using arraystar human long noncoding RNAs (lncRNA) microarray V3.0. Two thousand six hundred eighty-two lncRNAs (log fold- changes > 2.3) were detected. The scatter plot is a visualization method used for assessing the lncRNAs **(A)** and mRNAs **(B)** expression variations between left ventricular noncompaction (LVNC) and the control group. The values of the X and Y axes in the scatter plot are the averaged normalized signal values of the group (log2 scaled). The green lines are fold-change lines. **(C)** Differentially expressed lncRNAs and mRNAs in plasma of LVNC and the control were analyzed using hierarchical clustering. Hierarchical clustering analysis arranges samples into groups by expression level, “red” indicates high relative expression, and “green” indicates low relative expression.

Microarray expression analysis identified numerous lncRNAs and mRNAs whose expression varies between LVNC and the control group. In this study, we used a *t*-test to identify differentially expressed RNAs. According to the Benjamini Hochberg FDR method and the corrected *p-*value (FDR), differentially expressed lncRNAs and mRNAs were screened with Fold change ≥ 2.0, FDR <*0.0*5. Compared with the *p-*value filter, this method can better reduce the false positive rate. Two thousand six hundred eighty-two lncRNA (1,568 up and, 1,114 downregulated) and 2,083 mRNA (1,066 up and, 1,017 down) transcripts were identified in serum compared with the control group (log fold-change > 2.0). The top 10 upregulated lncRNA that were significantly downregulated were ENST00000550301, ENST00000433152, uc002nqf.1, ENST00000415393, ENST00000584722, TCONS_00029778, ENST00000443008, TCONS_00024700, NR_003256, ENST00000485225, TCONS_00006918, ENST00000423402, TCONS_00006919, uc022bqs.1, uc021ybu.1, ENST00000537478, and ENST00000447277. The differentially expressed top twenty lncRNA and mRNA were listed in Tables 3, 4. A Scatterplot is used for assessing the lncRNA expression variation between the two groups of LVNC and the control (Figures 1A,B). Hierarchical Cluster shows lncRNA expression patterns (Figure 1C).

**Table 3:** Differentially expressed long noncoding RNA (LncRNA).

**Seq name**	**Gene symbol**	**Fold change**	**P-value**	**Regulation**	**Chrom**	**Group-test**	**Group-control**
ENST00000550301	RP11-1100L3.7	63	1.26125E-07	up	Chr12	8.33	2.35
ENST00000433152	RP11-222A11.1	55	7.95746E-06	up	Chr10	8.74	2.94
uc002nqf.1	AX748435	49	1.83497E-07	up	Chr19	7.97	2.35
ENST00000415393	AC009518.5	47	1.7964E-05	up	Chr7	9.36	3.79
ENST00000584722	RP11-781P6.1	35	2.17895E-08	up	Chr18	7.57	2.46
TCONS_00029778	XLOC_014177	33	1.78035E-05	up	Chr22	8.25	3.21
ENST00000443008	RP5-1028L10.1	32.7	3.22805E-07	up	Chr1	7.38	2.35
TCONS_00024700	XLOC_011979	32.3	1.52019E-06	up	Chr16	7.91	2.90
NR_003256	ABCD4	31.2	2.50659E-05	up	Chr14	7.42	2.46
ENST00000485225	GSTTP2	29.7	0.0085	up	Chr22	11.29	6.40
TCONS_00006918	XLOC_002730	22.99	0.0005	down	Chr3	8.54	13.06
ENST00000423402	PCBP1-AS1	12.92	0.0003	down	Chr2	6.77	10.46
TCONS_00006919	XLOC_002730	12.92	0.0008	down	Chr3	10.09	13.78
uc022bqs.1	JA040725	11.76	0.0018	down	ChrM	4.42	7.98
uc021ybu.1	HM358988	9.24	0.0026	down	Chr5	5.89	9.1
ENST00000537478	RP11-407A16.3	8.82	0.0005	down	Chr12	6.95	10.09
ENST00000447277	RP11-626E13.1	8.64	0.0003	down	Chr4	3.96	7.07
ENST00000560512	RP11-566K19.4	8.25	0.0460	down	Chr15	6.15	9.19
TCONS_00006916	XLOC_002730	7.90	0.0069	down	Chr3	8.56	11.54
NR_024458	TPT1-AS1	7.67	0.0031	down	Chr13	3.67	6.61

**Table 4:** Differentially expressed mRNA.

**Seq name**	**Gene symbol**	**Fold change**	***P*** **-value**	**Regulation**	**Chrom**	**Group-test**	**Group-control**
ENST00000361205	C18orf1	133	8.66382E-07	up	Chr18	9.40	2.35
NM_005371	METTL1	67	6.73727E-05	up	Chr12	9.65	3.59
NM_018682	MLL5	65	6.41365E-06	up	Chr7	9.27	3.24
NM_057180	VPS29	61	1.75258E-05	up	Chr12	8.65	2.70
NM_016226	VPS29	52	2.08291E-05	up	Chr12	8.33	2.61
ENST00000399848	C18orf1	52	4.06472E-06	up	Chr18	10.12	4.43
NM_020061	OPN1LW	40	3.56748E-06	up	ChrX	7.68	2.35
NM_030774	OR51E2	39	1.25883E-06	up	Chr11	7.64	2.35
NM_020987	ANK3	38	2.9915E-07	up	Chr10	7.58	2.35
NM_176810	NLRP13	37	2.27229E-05	up	Chr19	7.55	2.35
ENST00000408934	PRR21	35	6E-13	down	Chr2	5.67	10.78
NM_001008388	CISD2	10	0.0002	down	Chr4	3.17	6.54
ENST00000328867	CAPN12	9.9	0.0009	down	Chr19	5.64	8.96
NM_001398	ECH1	7.8	7.36405E-05	down	Chr19	5.27	8.23
NM_001185100	CD22	7.01	0.04645	down	Chr19	3.79	6.60
NM_001136509	ZNF843	6.88	6.95865E-05	down	Chr16	5.66	8.44
NM_022166	XYLT1	6.789	9.88842E-05	down	Chr16	5.51	8.27
NM_001145093	ZNF619	6.71	0.0301	down	Chr3	5.85	8.59
NM_024102	WDR77	6.66	0.0024	down	Chr1	4.37	7.11
NM_006872	GTF2A1L	6.40	1.08375E-08	down	Chr2	2.71	5.39

### Real-Time Quantitative PCR Validation

**Figure 2:**
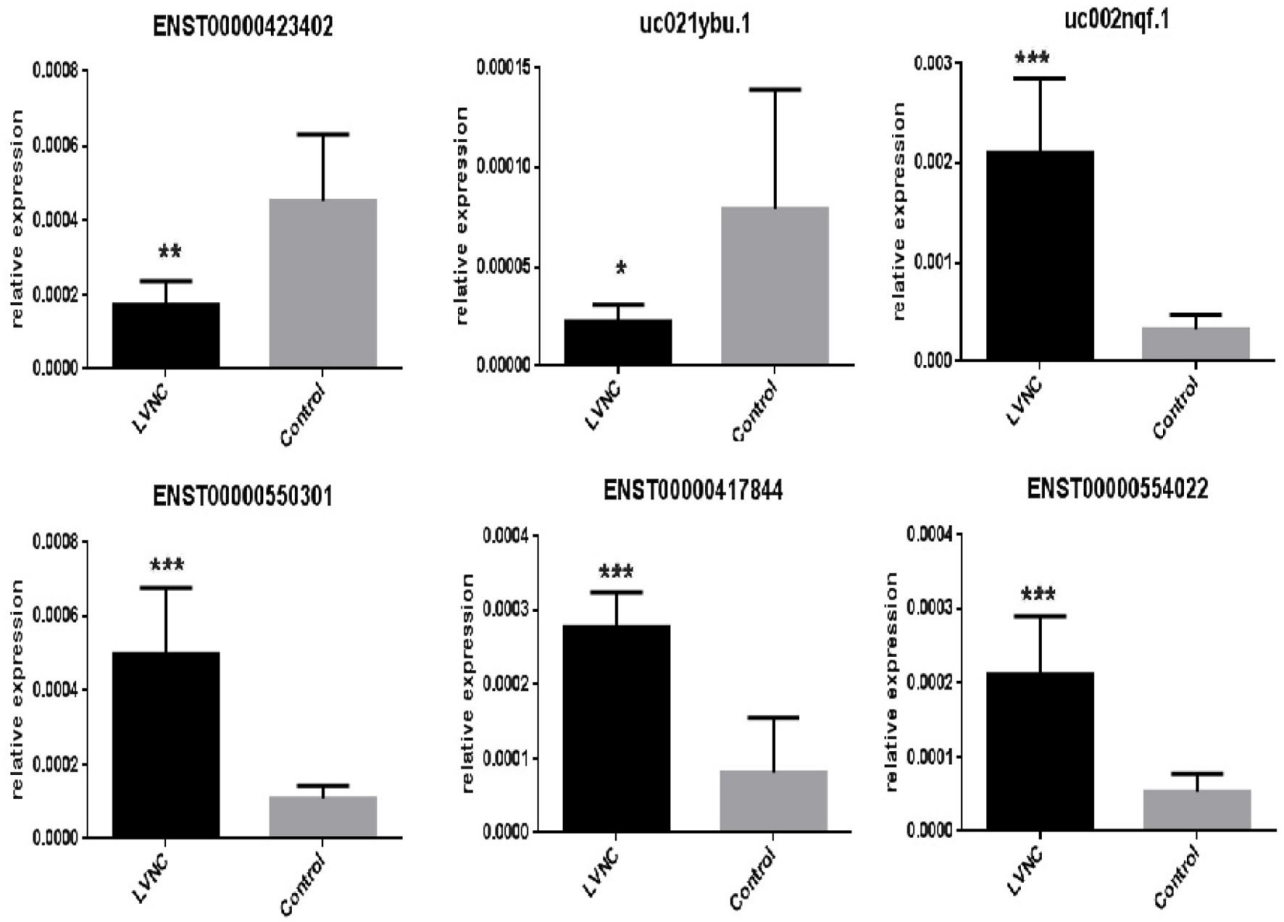
RT-QPCR validation. RT-QPCR was used to confirm the expression of six long non-coding RNAs, ENST00000423402, uc021ybu.1, uc002nqf.1, ENST00000550301, ENST00000417844, and ENST00000554022, in the LVNC and control groups. β-actin was used as an internal control. Values are presented as the mean ± standard error of the mean. ^*^p <0.05, ^**^p <0.01, ^***^p <0.001.

By using quantitative real-time reverse-transcription PCR (RT-QPCR), the top three up-regulated lncRNAs and top three downregulated lncRNAs with log fold-changes > 2 were selected to test and verify the microarray data in different blood samples of LVNC patients and paired healthy people. Due to the failure of TCONS_00006918 and TCONS_00006919 primer design, we selected the downregulated expression ENST00000423402, uc022bqs.1, and uc021ybu.1 for PCR verification. The downregulated expression of ENST00000423402, uc021ybu.1, and the upregulated expression of uc002nqf.1 and ENST00000550301 were statistically significant (*p* <0.05). Two lncRNAs (ENST00000417844 and, ENST00000554022) were randomly selected to prove the consistency of microarray and qPCR. The expression of lncRNA uc002nqf.1, ENST00000550301, ENST00000417844 and, ENST00000554022 was up-regulated, and ENST00000423402 and uc021ybu.1 was downregulated in the LVNC samples vs. the control samples (Figure 2), consistent with the microarray results. Thus, microarray data profiling indicated a series of lncRNAs that were constantly differentially expressed between LVNC blood and healthy blood.

### GO Analysis and Pathway Analysis

To establish the involvement of these RNAs in cellular pathways, we used GO databases to analyze their potential biological functions. As previously reported, GO analysis was performed to determine the gene and gene product enrichment, which covered three domains: biological processes (BP), cellular components (CC), and molecular functions (MF) (25).

**Figure 3:**
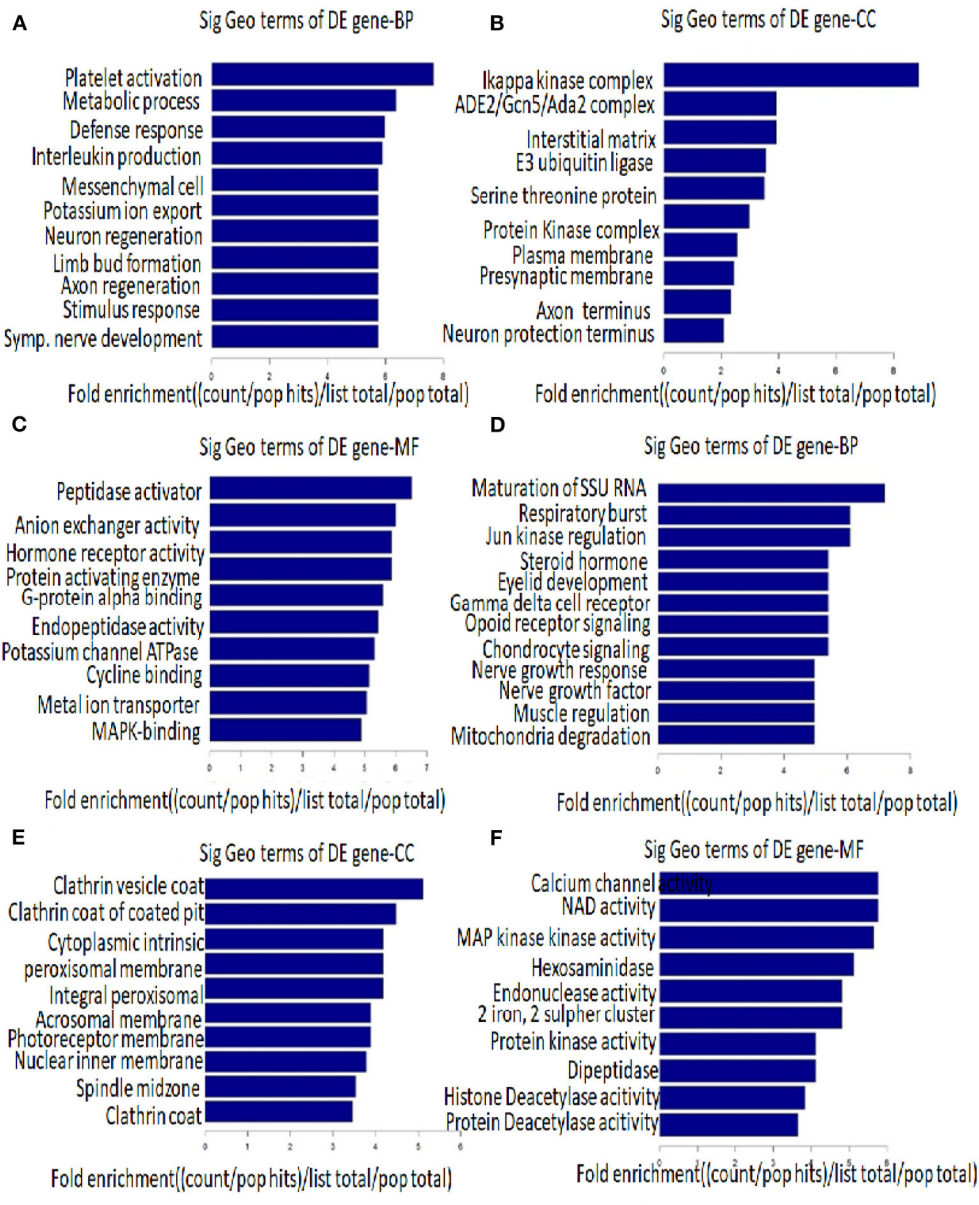
Gene ontology (GO) analysis. The top 10 GO terms associated with coding gene functions of upregulated lncRNAs **(A–C)** and downregulated lncRNAs **(D–F)** are listed.

**Figure 4:**
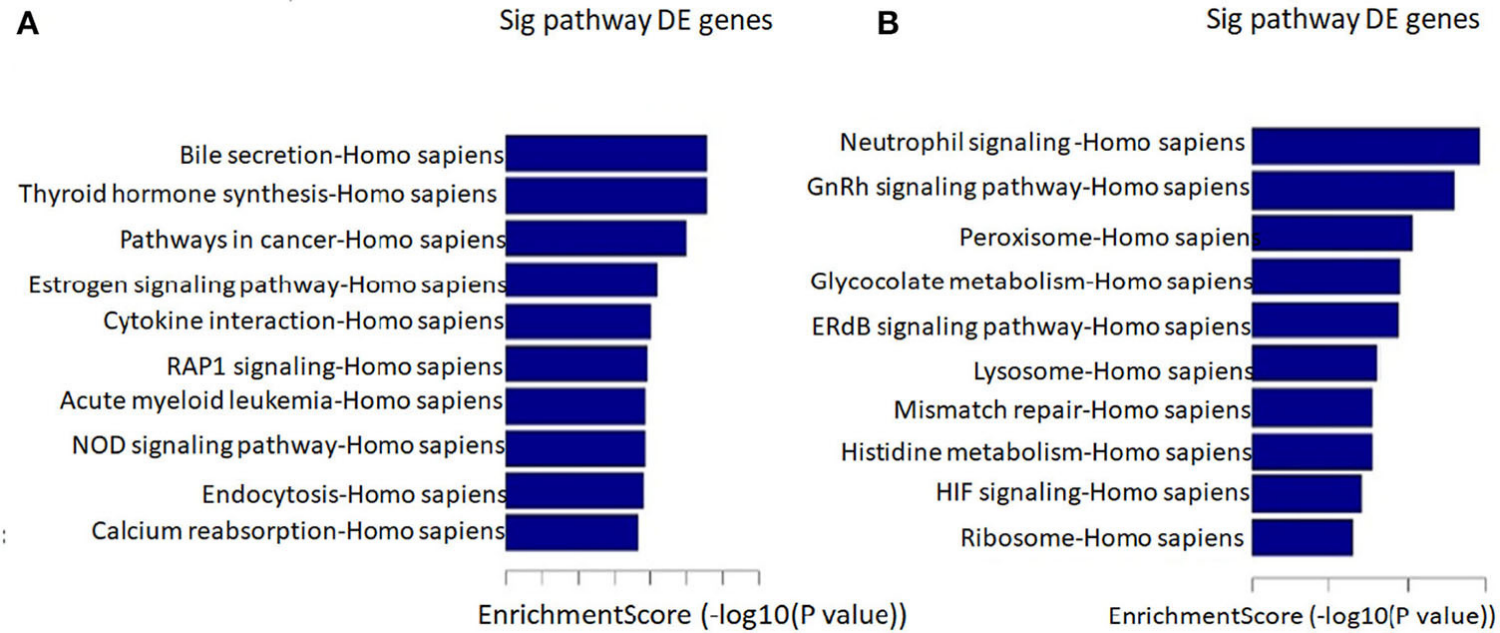
Pathway analysis. The top 10 pathways that associated coding gene of up-regulated lncRNAs **(A)** and downregulated lncRNAs **(B)** are listed.

We found that the highest GO classification targeted by underregulated transcripts were intracellular signal transduction (BP), plasma membrane (CC), and enzyme binding (MF) (Figures 3A–C). Meanwhile, the highest GO classification targeted by the overregulated transcripts were protein modification process (BP), an integral component of the plasma membrane (CC), and protein binding (MF), (Figures 3D–F). In addition, the KEGG pathway analysis demonstrated that the “bile secretion” and “thyroid hormone synthesis” were the most significant pathways containing differentially upregulated mRNAs. Increased bile secretion is shown to have an adverse effect and promotes heart failure in the long run and thus, may be relevant for developing LVNC (26, 27). Thyroid hormone disbalance has long been known to be a risk factor for heart diseases although its role in LVNC is not demonstrated (28, 29). The “neurotrophin signaling pathways” and “GnRH signaling pathways” were also important pathways containing differentially downregulated mRNAs (Figures 4A,B).

### GSEA Enriched Pathway

**Figure 5:**
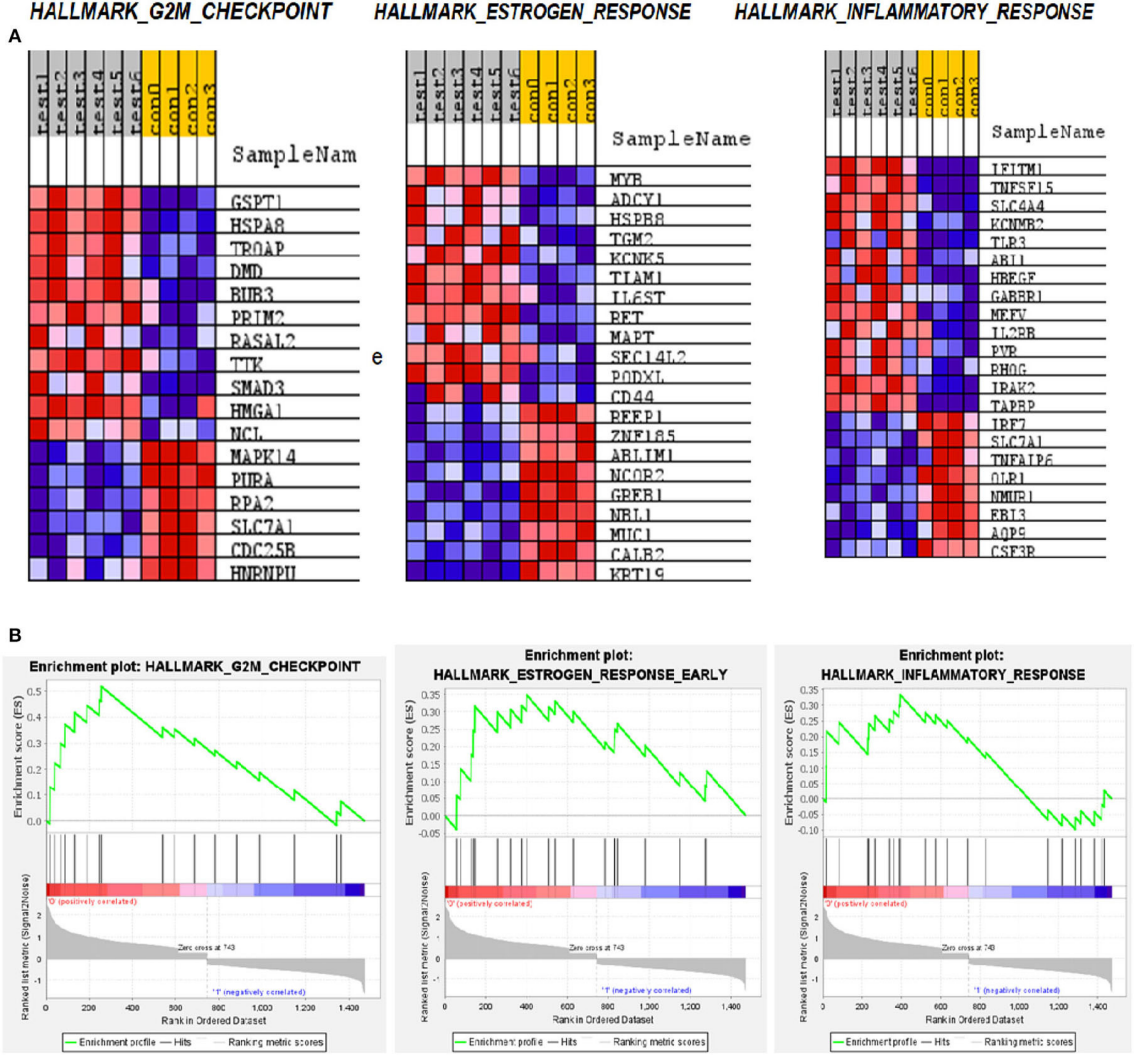
Gene set enrichment analysis (GSEA) pathways for differentially expressed mRNA in LVNC. **(A)** Heatmap of the three most enriched pathways showing genes. **(B)** Enrichment score of these pathways.

The Hallmark gene set with which GSEA has analyzed covers a wide range of pathways that would capture a set of genes represented in DEG. Our analysis with the top 1,500 genes identified 13 pathways that are enriched significantly. The rank order of genes with top enrichment score and their heatmap is shown in Supplementary Table 1 and Supplementary Figure 1. Among the pathways with the highest enrichment scores are the G2M transition pathway, Estrogen response pathway, and inflammatory pathway (Figure 5).

**Figure 6:**
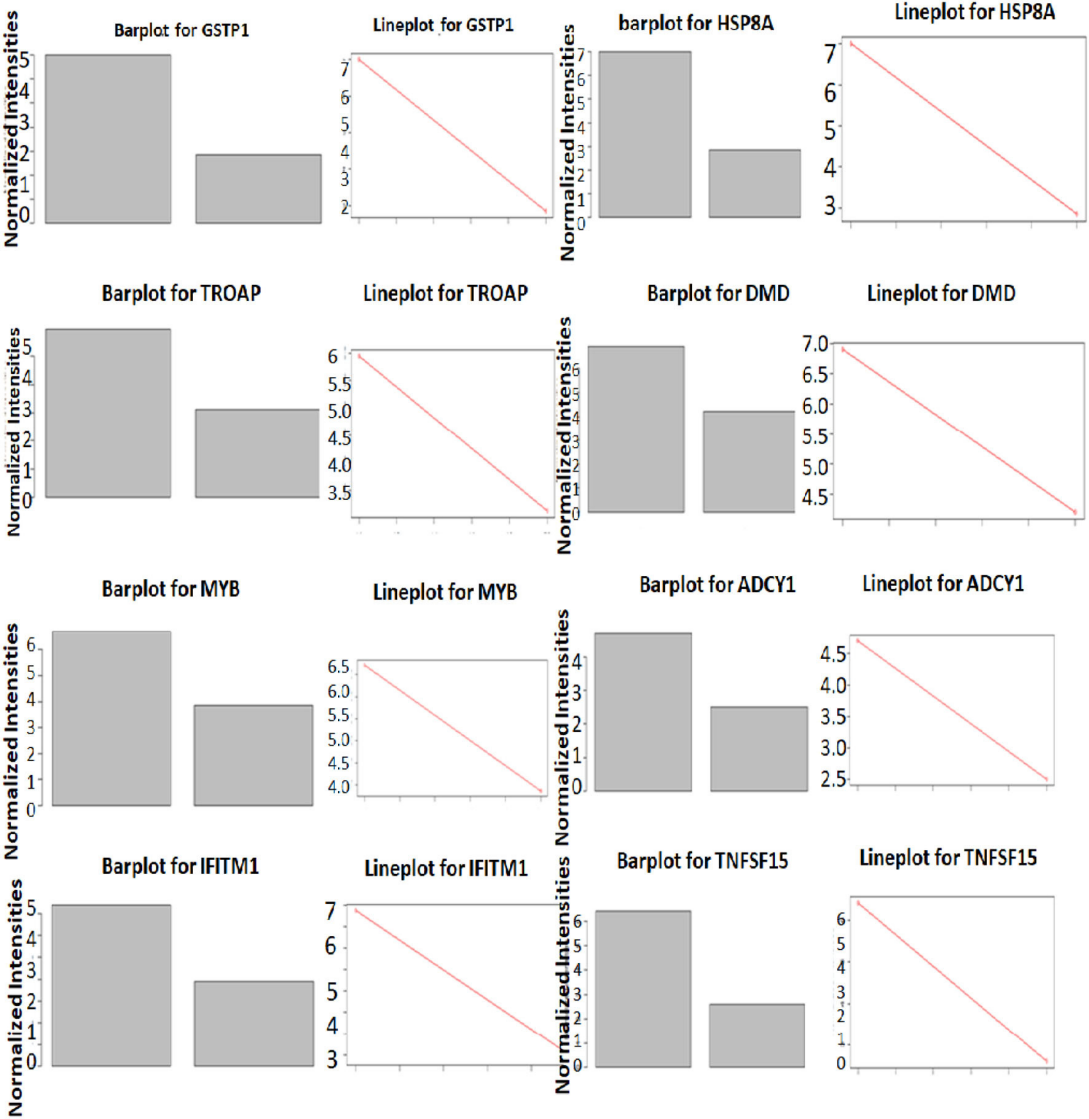
Differential expression of genes in LVNC and control in GSEA enriched pathways. Expression differences of top three genes in LVNC and healthy subjects in three enriched pathways.

The significance of the G2M pathway is relevant to the LVNC since many stress-responsive genes often play a role in heart development and pathogenesis (30). The glutathione-S-Transferase pi 1 (GSTP1) gene is highly polymorphic and involved in many diseases including cancer and heart failure (31). Its role in LVNC could be further investigated. HSPA8 (Hsc-70) is reported to be a titin interacting protein in an LVNC gene-edited mice model. Therefore, this gene could have a critical role in pathogenesis (32). Trophinin associated protein (TROAP) is shown to be involved in myocardial infarction (33), thus, its role in LVNC could also be important. Dystrophin (DMD) is known to play a role in LVNC development and pathogenesis (34), justifying its higher expression in patients (Figure 6). MYB is a nuclear protein and acts as a transcription factor for many cellular pathways in cardiac development (35). Adenylate cyclase (ADCY1) is involved in cardiac development and cardiomyopathy (36). HSPB8 is a heat shock protein and is also involved in cardiac development and cardiomyopathy (37). IFITM1 is an interferon-induced transmembrane protein that helps with the proliferation of neonatal cardiomyocytes in rats (38). TNF-inducing gene (TNFSF15) plays a critical role in neovascularization and inflammation in the heart. Thus, it could also play a critical role in developing LVNC (39).

### Biological Functions of DEGs

**Figure 7:**
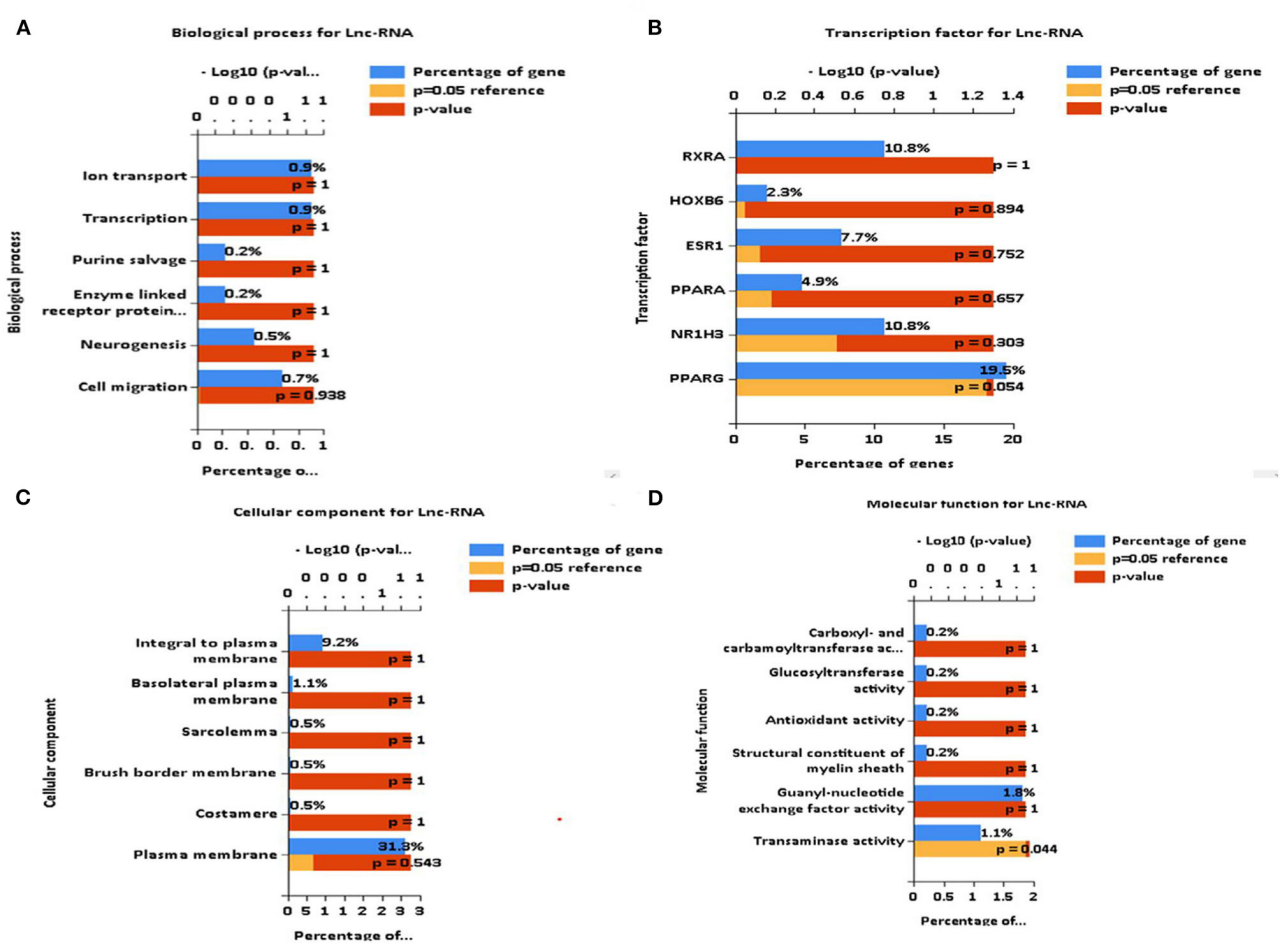
Identification of biological functions of differentially expressed genes (DEGs). **(A–D)** FUNRICH identified several critical biological functions ranging from cellular components to transcription factors.

**Figure 8:**
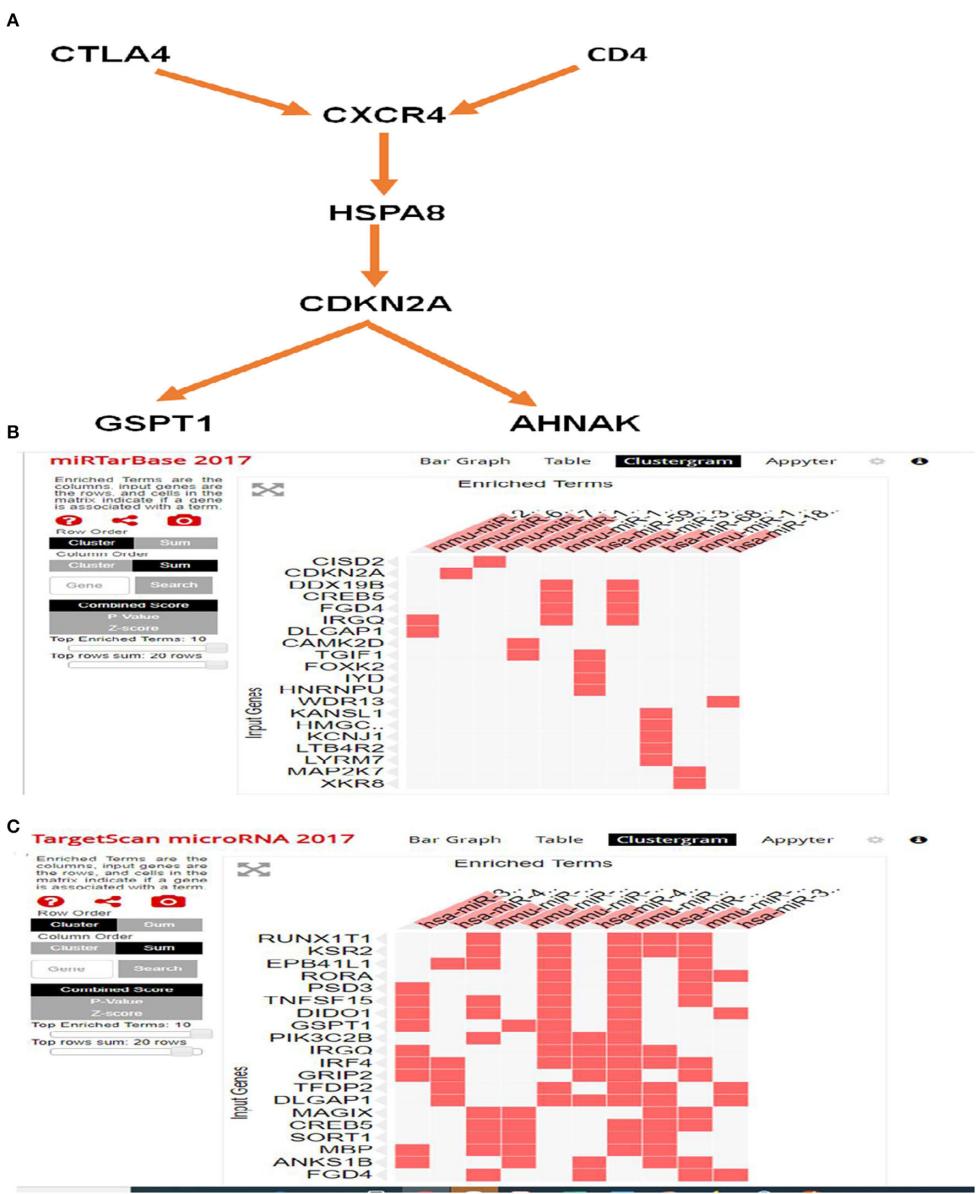
Interactome and miRNA target analysis using FUNRICH and EnrichR. **(A)** Major pathway includes CTLA4, CDKN2A, and HSPA8 involves cell cycle, apoptosis, and autophagy pathway. **(B)** Target miRNA analysis by miRTarbase that identifies CISD2 and CDKN2A. **(C)** Target miRNA analysis by targetmiRNAScan identifies RUNXIT1 and KSR2.

The biological functions of these DEGs are deduced as components, such as biological processes, transcription factors, cellular components, and molecular functions. Major biological processes include ion transport, transcription, purine cleavage, neurogenesis, and cell migration (Figures 7A–D). Interactome analysis identified a major pathway (Figure 8A) that includes CTLA4, CDKN2A, and HSPA8 which denotes cell cycle, apoptosis, autophagy, and cellular remodeling (40).

### DEGs Modulate a Group of Targeted MiRNAs

Analysis with EnrichR identified several important pathways (Supplementary Figure 2) such as transcription factor (TF) perturbation, epigenetic genes, and more importantly, miRNA. It used the miRtargetBase 2017 and targetScanmicroRNA2017 database and identified miRNAs that are linked to these DEGs (Tables 5, 6). miRTargetBase miRNAs identified CISD2, CDKN2A, etc. (Figure 8B) as target genes, and miRTargetScan identified RUNXT1 and KSR2 (Figure 8C) as target genes. Their role in developing LVNC could be further established.

**Table 5:** Identification of target miRNA of differentially expressed genes (DEGs) (miRtargetBase 2017).

**Index**	**Name**	***P*** **-value**	**Adjusted** ***P*****-value**	**Odds ratio**
1	mmu-miR-770-5P	0.011	0.95	16.16
2	mmu-miR-202-5P	0.011	0.95	16.16
3	mmu-miR-146a-3p	0.011	0.95	16.16
4	mmu-miR-615-5p	0.011	0.95	16.16
5	mmu-miR-1907	0.0028	0.90	7.72
6	hsa-miR-597-3p	0.0018	0.90	6.34
7	mmu-miR-322-5p	0.0038	0.90	7.05
8	hsa-miR-6870-3p	0.00041	0.90	4.94
9	mmu-miR-1224-5p	0.023	0.95	10.10
10	hsa-miR-181a-3p	0.023	0.95	10.10

**Table 6:** Identification of target miRNA of DEGs (targetScanmicroRNA2017).

**Index**	**Name**	***P*** **-value**	**Adjusted**	**Odds**	**Combined**
			***P*** **-value**	**ratio**	**score**
1	hsa-miR-100	0.75	0.80	0.80	0.23
2	hsa-miR-103b	0.0042	0.070	1.51	8.27
3	hsa-miR-1180	0.38	0.56	1.12	1.07
4	hsa-miR-1181	0.74	0.80	0.76	0.23
5	hsa-miR-1203	0.089	0.29	1.38	3.33
6	hsa-miR-1204	0.0064	0.091	1.67	8.41
7	hsa-miR-1225-5p	0.083	0.29	1.25	3.12
8	hsa-miR-1234	0.026	0.19	1.39	5.03
9	hsa-miR-1244	0.22	0.43	1.14	1.70
10	hsa-miR-1247	0.0022	0.051	1.80	10.94

The top significant miRNAs, hsa-miR-3197 and hsa-miR-6780-3p are identified by miRTargetbase and miRTargetScan, respectively. The function of Has-miR-3197 has not been researched extensively though it has been reported to be a potential biomarker for diabetic retinopathy (41). However, its role in cardiovascular disease and LVNC pathogenesis could be investigated. The has-miR-6780-3p is shown to be downregulated in the plasma of patients with increased low-density lipoprotein (LDL) which could have an impact on developing cardiac dysfunction (42).

## Discussion

Although noncompaction of the myocardium is seen in association with other congenital cardiac abnormalities, it can also occur as a primary disorder in the absence of other structural heart diseases (3). It remains unclear whether it represents a distinct disease or a morphological trait shared by many phenotypically different cardiomyopathies (43). Samples selected for this study were isolated cases of left ventricular myocardium which appears in a variety of clinical manifestations, including heart failure, thromboembolic events, and tachyarrhythmias. The aim of this study was to determine the differently expressed lncRNA in the blood of LVNC and to provide the detectable biomarkers for clinical diagnosis. These detectable biomarkers may provide information for further research on regulation during the myocardium noncompaction process through LncRNA and coding gene analysis.

Long noncoding RNA (LncRNA) and coding gene analysis showed that one upregulated lncRNA AC068542.1 upregulates the expression of the mRNA-DPP10. DPP10 encodes a single-pass type II membrane protein that is a member of the S9B family in clan SC of the serine proteases. It binds specific voltage-gated potassium channels and alters their expression with biophysical properties. Turnow K. et al. reported that the interaction of DPP10a, expressed in the human atrium with Kv4.3 channels, generates a sustained current component of Ito, which may affect the late repolarization phase of atrial action potentials (44). Thus, the modulated expression of this gene could have an impact on LVNC pathogenesis.

AC068542.1 lncRNA has been implicated in modulating transcription factors and has a distinct epigenetic mark at its transcription start site (TSS). The author suggested an alternative role of its regulation (45). This lncRNA is also implicated in the modulation of miRNA in breast cancer (46), however, its role in LVNC is not clear.

Another upregulated lncRNA RP11-973D8.4 downregulates the adjacent gene CDK2, which encodes a member of a family of serine/threonine protein kinases that participates in the cell cycle regulation. The activity of this protein is especially critical during the G1 to S phase transition. Spencer SL et al. found that cells at the end of mitosis decide whether or not to start the next cell cycle by immediately activating CDK2 or by entering a transient G0-like state by suppressing CDK2 activity (47).

A differentially expressed top mRNA is the METTL1 (methyltransferase 1) gene which has a binding motif of S-S-adenosylmethionine binding motif. Methyltransferase mediates the formation of N(7)-methylguanine in a subset of RNA species, such as tRNAs, mRNAs, and microRNAs (miRNAs) (PubMed:12403464, PubMed:31031084, PubMed:31031083) and catalyzes the formation of N(7)-methylguanine at position 46 (m7G46) in tRNA (48). METTL1 gene promotes VEGFA mRNA translation that has implications in heart development (49).

Gene Ontology and KEGG pathway analysis identified the “bile acid secretion pathway” and “Thyroid hormone synthesis pathway” that are known to have a profound impact on heart diseases. However, the roles of these pathways have not been extensively studied in relation to the development of LVNC (27, 28). Furthermore, GSEA analysis identified HSPA1, DMD, and ADCY1 as having significant relevance in developing heart ailments and which presumably play a critical role in developing LVNC (32, 34, 36).

Finally, we identified DEG-specific target miRNAs that may play a critical role in the pathogenesis of LVNC but they have not been investigated extensively. It is worthwhile to further investigate these miRNAs and their target genes to explore their role in LVNC pathogenesis. To further confirm the roles of these genes in patients with LVNC, animal models and gene editing techniques, such as CRISPR-Cas9, could effectively discover mechanisms underlying this disease and, thus, shed light on potential novel targeting strategies for treating patients with LVNC.

## Data Availability Statement

The original contributions presented in the study are included in the article/Supplementary Material, further inquiries can be directed to the corresponding author/s.

## Ethics Statement

The studies involving human participants were reviewed and approved by Institutional Review Board (IRB) of First Affiliated Hospital of Nanchang University. The patients/participants provided their written informed consent to participate in this study.

## Author Contributions

ZZ and HW: conceptualization and funding acquisition. QT, HN, DL, AM, ZZ, and HW: methodology. QT, HN, DL, NT, QY, VN, YW, AM, ZZ, and HW: validation. QT, HN, DL, NT, VN, AM, ZZ, and HW: writing and editing. All authors contributed to the article and approved the final manuscript.

## Funding

This study was supported by a grant from the National Nature Science Foundation of China (No. 30960119, 81260044), Science and Technology Planning Project of Jiangxi Provincial Health Commission (No. 20195129), and Natural Science Foundation of Jiangxi Province (No. 20202ACBL206002).

## Conflict of Interest

The authors declare that the research was conducted in the absence of any commercial or financial relationships that could be construed as a potential conflict of interest.

## Publisher's Note

All claims expressed in this article are solely those of the authors and do not necessarily represent those of their affiliated organizations, or those of the publisher, the editors and the reviewers. Any product that may be evaluated in this article, or claim that may be made by its manufacturer, is not guaranteed or endorsed by the publisher.
