# Supplementary material for: Expression Signatures of Long Noncoding RNAs in Left Ventricular Noncompaction
Source: Front Cardiovasc Med. 2021 Nov 10;8:763858. doi: 10.3389/fcvm.2021.763858 (PMC8631435; doi:10.3389/fcvm.2021.763858)
Supplement: Supplementary file 1 [file Data_Sheet_1.docx]

Supplementary Material
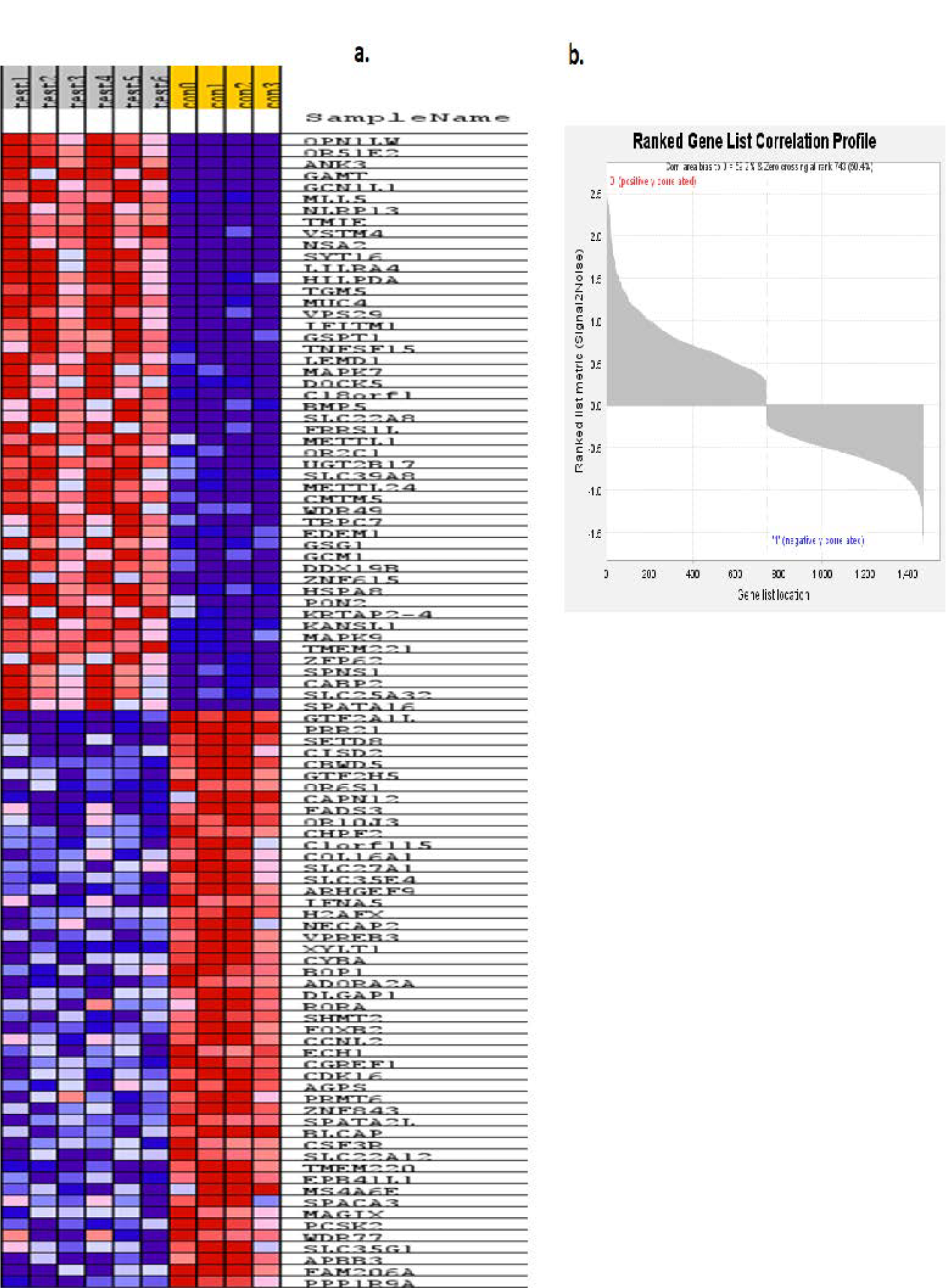


**Supplementary Figure1. Heatmap of rank order of enrichment genes.**

**
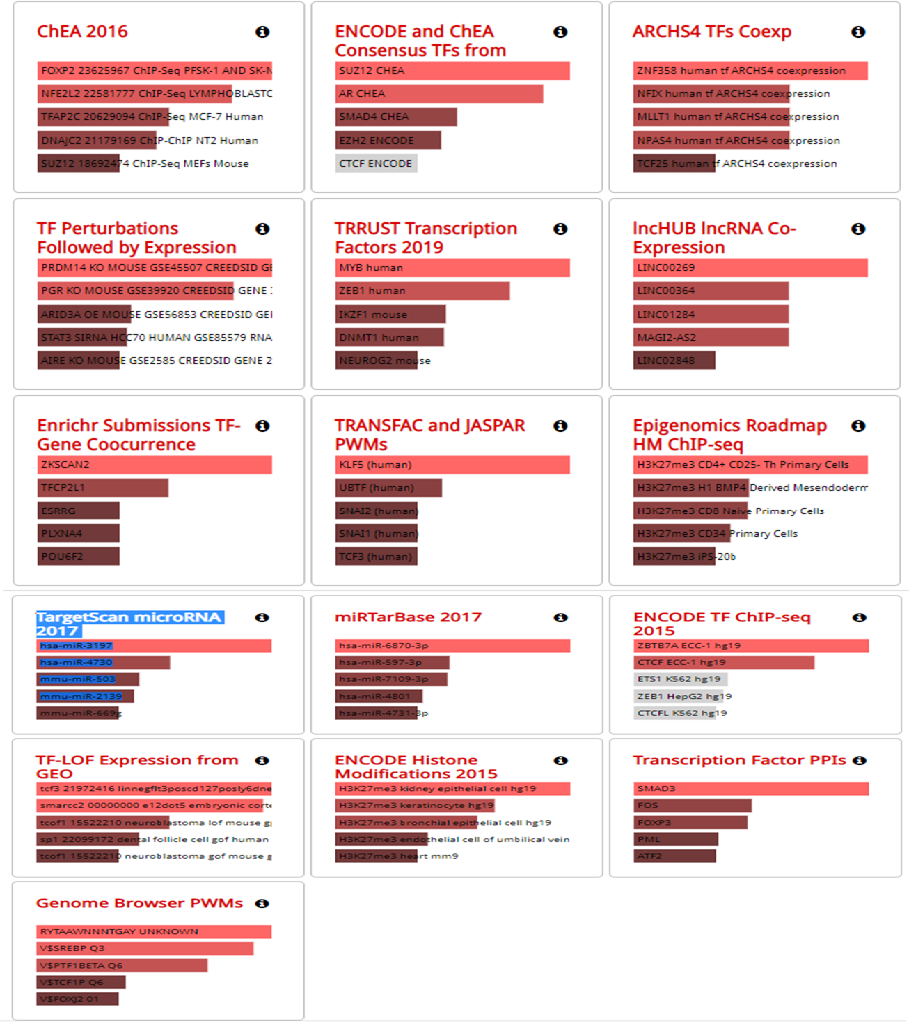
**

**Supplementary Figure 2. Funrich identified various biological functions of DEGs.**

**Supplementary table 1**: Enriched pathway rank order of 99 genes.

| **NAME** | **ENRICHMENT SCORE** |
| --- | --- |
| OPN1LW | 2.6602738 |
| OR51E2 | 2.6503518 |
| ANK3 | 2.636782 |
| GAMT | 2.457405 |
| GCN1L1 | 2.4160337 |
| MLL5 | 2.3892035 |
| NLRP13 | 2.3780003 |
| TMIE | 2.3703957 |
| VSTM4 | 2.3394392 |
| NSA2 | 2.3334827 |
| SYT16 | 2.309594 |
| LILRA4 | 2.2790363 |
| HILPDA | 2.2719035 |
| TGM5 | 2.258921 |
| MUC4 | 2.2583334 |
| VPS29 | 2.2212925 |
| IFITM1 | 2.2102768 |
| GSPT1 | 2.202665 |
| TNFSF15 | 2.1423354 |
| LEMD1 | 2.1195233 |
| MAPK7 | 2.092 |
| DOCK5 | 2.0395873 |
| C18orf1 | 1.96901 |
| BMP5 | 1.958839 |
| SLC22A8 | 1.9559479 |
| FRRS1L | 1.896788 |
| METTL1 | 1.8596703 |
| OR2C1 | 1.8528402 |
| UGT2B17 | 1.8367251 |
| SLC39A8 | 1.8122046 |
| METTL24 | 1.7765083 |
| CMTM5 | 1.7760447 |
| WDR49 | 1.7554382 |
| TRPC7 | 1.7538061 |
| EDEM1 | 1.7122577 |
| GSG1 | 1.7069877 |
| GCM1 | 1.700911 |
| DDX19B | 1.6830304 |
| ZNF615 | 1.677452 |
| HSPA8 | 1.6372328 |
| PON2 | 1.6368212 |
| KRTAP2-4 | 1.6101612 |
| KANSL1 | 1.6038004 |
| MAPK9 | 1.6035903 |
| TMEM221 | 1.574899 |
| ZFP62 | 1.5737362 |
| SPNS1 | 1.568894 |
| CABP2 | 1.5618242 |
| SLC25A32 | 1.5552593 |
| SPATA16 | 1.5439702 |
| SLITRK3 | 1.5393786 |
| EEFSEC | 1.5317029 |
| ZXDC | 1.5285225 |
| ENPP2 | 1.5279971 |
| DDO | 1.5258592 |
| SYNDIG1L | 1.5132465 |
| MORN4 | 1.4903282 |
| ACVR1C | 1.4837177 |
| MYB | 1.4759712 |
| HMGXB3 | 1.4653934 |
| ARL6IP4 | 1.4637631 |
| ADSSL1 | 1.4582072 |
| GSTZ1 | 1.4504341 |
| CXCL14 | 1.4472635 |
| CENPN | 1.44302 |
| AGAP7 | 1.4403282 |
| TROAP | 1.4220889 |
| OBSCN | 1.4181123 |
| ITGAM | 1.4094723 |
| SNX18 | 1.3959566 |
| CACNA1G | 1.3901362 |
| LYRM7 | 1.3818654 |
| PPP4R1 | 1.3810419 |
| KANK1 | 1.375932 |
| PPP6R3 | 1.3713651 |
| SEC14L4 | 1.3695478 |
| ADCY1 | 1.368233 |
| DNASE1 | 1.3637005 |
| PAOX | 1.3578109 |
| ALPK2 | 1.3437854 |
| SLC4A4 | 1.3386437 |
| KIAA0494 | 1.3377475 |
| ASGR1 | 1.334036 |
| ARIH2 | 1.332287 |
| TP53AIP1 | 1.3299587 |
| WFDC9 | 1.3282876 |
| MCF2 | 1.3241143 |
| DMD | 1.3202555 |
| SLC35A5 | 1.3171312 |
| MDH1B | 1.3150628 |
| HOXB6 | 1.3149848 |
| OSBPL7 | 1.3029398 |
| SLC12A6 | 1.2965502 |
| CD4 | 1.2836585 |
| KLF1 | 1.2815231 |
| C12orf57 | 1.2802111 |
| ZMYND11 | 1.2797409 |
| KCNE2 | 1.2776265 |
| CEACAM21 | 1.2708935 |
